# The redistributive power of cash transfers vs VAT exemptions: A multi-country study

**DOI:** 10.1016/j.worlddev.2021.105742

**Published:** 2022-03

**Authors:** Ross Warwick, Tom Harris, David Phillips, Maya Goldman, Jon Jellema, Gabriela Inchauste, Karolina Goraus-Tańska

**Affiliations:** a Institute for Fiscal Studies, United Kingdom; b Commitment to Equity Institute, United States; c The World Bank, United States; d University of Warsaw, Poland, and the World Bank, United States

**Keywords:** Value-added tax, Cash transfers, Taxation, Inequality, Redistribution, Microsimulation

## Abstract

Like high-income countries, low- and middle-income countries (LMICs) offer reduced rates and exemptions on particular goods and services in their value-added tax (VAT) systems. These policies are often motivated by distributional concerns and target items thought to take up a larger share of the budgets of poorer households. This paper explores the effectiveness of such policies in six LMICs. We estimate their impact on tax revenues, inequality and poverty, and compare these effects to existing cash transfer schemes and a hypothetical Universal Transfer (UT) funded by broadening the VAT base. To do so, we use tax-benefit microsimulation models incorporating input–output tables, allowing us to estimate the impact of exemptions on consumer prices due to VAT embedded in supply chains. We show that although preferential VAT rates reduce poverty, they are not well targeted towards poor households overall. Existing cash transfer schemes are better targeted but generally have limited coverage. A UT funded by a broader VAT base would create large net gains for the poorest households, reducing inequality and most measures of extreme poverty in each of the countries studied. Our results suggest that the widespread practice of providing special VAT treatment to certain goods and services is an expensive way of reaching poor households. In principle, expanding the VAT base and social protection schemes in tandem has the potential to both raise tax revenues and reduce poverty. Such reforms therefore warrant consideration for LMICs as they pursue Domestic Revenue Mobilisation and broader development objectives.

## Highlights

• We use microsimulation models to study the effects of reduced VAT rates and exemptions on revenues, poverty and inequality.
• In isolation, reduced VAT rates and exemptions in six LMICs studied are poverty-reducing.
• They are often expensive in terms of foregone tax revenue, and in all cases richer households benefit the most in cash terms.
• Though better targeted at poor households, existing cash transfer programmes often miss large shares of poor households.
• Recycling the revenue yield from a broader VAT base with universal benefits would reduce inequality and most measures of poverty.

## 1 Introduction

Low- and middle-income countries (LMICs) are characterised by higher degrees of poverty and inequality than high-income countries (e.g. Chen and Ravallion, 2010, Lakner and Milanovic, 2016). While taxes and transfers can be a powerful means of affecting the income distribution, governments in developing countries are more constrained by both administrative capacity and overall resources in terms of the policy options available (Goñi, López and Servén, 2011). One common approach is to try to target poorer households through what they buy: for instance, most value-added tax (VAT) systems offer exemptions or lower tax rates (hereafter “preferential VAT rates”) on certain types of consumption. Despite the pervasiveness of this approach, there is little existing evidence as to how well targeted such policies are, and how alternatives compare.

Evidence from the EU (IFS, 2011) and OECD (OECD/KIPF, 2014) suggests that VAT systems are a poor way to redistribute resources. However, in LMICs home production, payment in-kind and (untaxed) informal sector purchases are important consumption sources (Deaton and Grosh, 2000, Bachas et al., 2021); VAT structures are often characterised by a broader base (Ebrill et al., 2001) which may mean the more limited number of preferential VAT rates are better targeted; and available policy options for redistribution are generally limited (Bastagli, Coady, & Gupta, 2015). Thus, existing findings do not necessarily carry over to such contexts.

This paper asks whether preferential VAT rates are an effective tool for supporting poor households in LMICs. Using household consumption data and microsimulation models for six LMICs, we study the effect of preferential VAT rates on the purchasing power of households, as well as their overall implications for tax revenues and measures of poverty and inequality. We contrast these effects with alternative policy options by comparisons with both real and hypothetical cash transfer programmes in each country.

Our empirical approach utilises sophisticated tax and benefit microsimulation models assembled for six countries: Ethiopia, Ghana, Senegal, Sri Lanka, Uzbekistan and Zambia. These combine detailed consumption data and input–output tables with a mapping of the statutory tax and benefit system, allowing us to estimate the impact of existing policy and hypothetical reforms under the assumption of unchanged behaviour and full incidence of taxes and transfers on households. Using these standard assumptions from the applied welfare analysis literature, and accounting for the impact of unrecoverable input VAT caused by VAT exemptions, we estimate the effect of existing preferential VAT rates on measures of household-level consumption and their effect on headline measures of poverty and inequality. We compare these with the effects of existing and hypothetical cash transfer schemes for which we either observe payment receipts in survey data or can simulate receipts using defined eligibility criteria.

Our first set of results considers the impact of preferential VAT rates in isolation for each country, excluding a common set of exemptions put in place for administrative reasons: those on public, health, education and financial services, domestic rents and small traders. We highlight three main findings. Firstly, these preferential rates constitute a substantial cost to the government in revenue terms (hereafter a tax expenditure or a VAT expenditure). For instance, in Sri Lanka a broader base could increase overall VAT revenue by up to 50%. Secondly, taken in isolation, these tax expenditures are likely to contribute significantly to poverty reduction, reducing the extreme poverty gap by up to 1.26 percentage points in Senegal, for instance. However, despite this, preferential VAT rates are a poor way of targeting resources towards poorer households. In all countries, the cash subsidy afforded by preferential VAT rates accrues largely to high consumption households, even when progressive as a proportion of consumption. In Ethiopia, Ghana and Zambia, they are not even progressive in proportional terms.

The rest of the paper considers alternative means of channelling resources towards poorer households, focusing on cash transfer programmes. While the countries we cover do not have the suite of targeted tax credits and benefit programmes available in many richer countries, they do all have some form of cash transfer programme aimed at poverty reduction. We show that these programmes are much better targeted towards low-consumption households than preferential VAT rates. However, their eligibility requirements often limit their scale compared to existing VAT expenditures, suggesting existing schemes would not always be suitable for large-scale compensation for VAT base broadening. They are also, in their current form, often donor-led and donor-financed, and do not necessarily carry the broad political support of the population (Opalo, 2021).

In the final stage of our analysis, we conduct reform simulations focusing on the simplest possible compensation scheme for a broader VAT base. In each country, we simulate the effect of broadening the VAT base and recycling the revenue raised in the form of a Universal Transfer (UT). In doing so, we maintain the aforementioned exemptions that are in place largely for administrative rather than distributional reasons. The scale of redistribution theoretically possible from such a reform depends on three factors: the overall cost of existing preferential VAT rates; the relative progressivity of these preferential rates; and the overall scale of consumption inequality. Our results show that given these factors, even an untargeted cash transfer scheme would be significantly more effective at supporting poorer households in every country that we study. The potential effects are large: for instance, if all additional revenue was recycled, we estimate a reduction in the extreme poverty headcount ranging from 0.13 percentage points in Uzbekistan to 1.10 percentage points in Ghana.

This paper contributes to a number of different strands of literature. Firstly, we add to a broad literature on the effects of tax and transfer policy on poverty and inequality in developing countries. Existing research consistently highlights the more limited redistributive impact of fiscal policy in LMICs, driven by both the more limited scale of tax and spending policies and differences in their composition, including the more limited coverage of progressive income taxes (Bastagli et al., 2015, Goñi et al., 2011, Inchauste and Lustig, 2017, Lustig, 2016). Preferential VAT rates are a pervasive feature of redistributive tax policy in LMICs, yet while the basis for such policies has been discussed theoretically (e.g. Keen, 2014) there is little existing empirical work outside of single country studies (e.g. Jansen and Calitz, 2017, van Oordt, 2018 – both for South Africa).^1^ By generating evidence from a number of LMICs using a consistent methodology, we shed new light on the distributional effects of preferential VAT rates in developing economies and, crucially, consider the potential for alternative policies to be more effective. This work is close in spirit to that of Del Granado, Coady and Gillingham (2012), who study who benefits from fuel subsidies in different developing countries and account for the indirect effect of subsidies enjoyed by producers, finding that the vast majority of such benefits accrue to richer households. More recently, Bachas, Gadenne and Jensen (2021) also study the design of VAT systems in developing countries, including the equity motivation for preferential rates on certain purchases. We differ in our main focus, however: whereas their research considers the implications of variation in the propensity to purchase from the informal sector across the consumption distribution for VAT design, we focus on modelling existing de jure tax systems and alternative social protection reforms.

More generally, we relate to papers studying the design of tax systems in developing countries, much of which has focused on VAT specifically in recent years given its ubiquity and its importance for revenue collections in such contexts (Keen, 2013, Cnossen, 2015). Much of the recent literature has focused on issues of VAT administration, including the drivers of VAT compliance and the role of specific interventions (Pomeranz, 2015, Naritomi, 2019, Waseem, 2020). Other research has considered the efficiency implications of VAT in LMICs, such as the impact of effective exemptions from VAT on firm sourcing decisions and informality (De Paula and Scheinkman, 2010). In contrast, this paper is concerned primarily with the equity implications of the design of VAT, focusing on the targeting of preferential rates that do not have a strong case for holding exempt status on administrative grounds. We show that on top of the potential distortive effects of exemptions, these policies lead to significant sums of foregone revenue and mostly benefit richer households.

Methodologically, we build on previous research that incorporates the effect of taxes levied on intermediate inputs on consumer prices – a consideration that is often overlooked in applied analyses of tax burdens at the household level. In our context, this effect arises through VAT exemptions which prohibit the reclaim of input taxes by producers. We estimate this effect using input–output tables and assumptions about the pass-through of VAT. Early work that uses input–output tables to study indirect tax reform includes that of Hughes (1986) and the effective tax rate model of Ahmad and Stern (1984). More recently, Coady (2008) (see also Inchauste and Jellema, 2018) illustrated how VAT exemptions in particular can be studied with similar methods; Giesecke and Hoang Nhi (2010) also incorporate this feature into macroeconomic modelling of VAT reform in Vietnam. Our contribution is to modify the assumptions of existing methods such that the full distributional (and tax revenue) implications of VAT changes can be estimated using only data on household consumption and input–output relationships. In particular, we do this by allowing for differential taxation and pricing of imported and domestic varieties of goods. We also describe how, in principle, the method that we set out could be extended to incorporate different effective tax rates by household or by firm, if and where more detailed data were available.

Our findings are of clear relevance to policymakers. As of 2020, 170 countries around the world had a VAT (OECD, 2020), with the tax contributing a larger proportion of total tax revenue in LMICs, and preferential rates are a ubiquitous feature of such systems. Raising additional tax revenue (“Domestic Revenue Mobilisation”, or DRM) is a central part of successfully achieving the Sustainable Development Goals, and the need for greater tax collection has only been heightened by the public finance and human development impacts of the COVID-19 crisis. Our findings suggest that broadening VAT bases may be a means of increasing revenues without increasing poverty if accompanied by the expansion of social protection programmes. The six countries that we study differ in terms of inequality, income level and VAT structure, lending some generalisability to our results. While we cannot claim that our findings will extend to every LMIC context, the strength of our conclusions suggest that the overall message is likely to apply in many countries.

This does not mean that governments in LMIC counties, including those of the countries in our study, should immediately abolish most preferential rates of VAT and introduce a UT. While we consider the merits of differential VAT rates on the grounds of redistribution, they can also be motivated on economic efficiency grounds in certain circumstances,^2^ although the consensus among public finance economists is that on both dimensions a broad base and uniform rate of VAT is likely to be superior (Ebrill et al., 2001). An increase in the average rate of VAT applied to consumption as a result of abolishing existing preferential rates and the introduction of a UT could also affect consumption and labour supply decisions, which are held fixed in our modelling, potentially affecting both households’ real incomes and tax revenues. And there are also many considerations for scaling up social protection programmes that are beyond the scope of this paper.^3^ As discussed later, the strength and consistency of our findings mean we are confident that our overall conclusions are robust, but governments would wish to consider these additional factors in a full economic impact assessment before implementing policy changes.

The rest of the paper proceeds as follows. In Section 2 we provide an overview of the VAT structure and direct cash transfer schemes in the six countries included in the analysis. We discuss our approach to modelling VAT and cash transfers in Section 3. 4, 5, 6 present our results from each country on existing preferential rates, existing cash transfer schemes, and a hypothetical reform that broadens the VAT base and introduces a UT, respectively. We conclude with a discussion of the findings from Sections 4–6, while also highlighting possible extensions to the analysis.

## 2 VAT and cash transfer systems in six LMICs

This section sets out the key features of the VAT systems and cash transfers that we model in Ethiopia, Ghana, Senegal, Sri Lanka, Uzbekistan and Zambia. We describe the main rules in place for the year of analysis for each country (see Table 2.1), with Appendix A providing further detail.

**Table 2.1:** Tax revenues and VAT revenues six LMICs.

**Country (Year)**	**Tax/GDP**	**VAT/GDP**	**VAT/Tax**
Ethiopia (2015–16)	11.5%	3.6%	31.1%
Ghana (2016)	11.3%	3.2%	28.0%
Senegal (2015)	16.4%	5.9%	35.8%
Sri Lanka (2017)	12.5%	3.3%	26.6%
Uzbekistan (2018)	17.4%	6.9%	39.4%
Zambia (2015)	14.4%	4.5%	31.2%

Source: Revenue data comes from national sources. GDP data comes from the World Bank and IMF.

### 2.1 The structure of VAT

The standard VAT rate varies from 15% in Ethiopia and Sri Lanka to 20% in Uzbekistan, with rates of 16% in Zambia, 17.5% in Ghana and 18% in Senegal in between.^4^ There are significant differences in the coverage of VAT, however: for instance, the household surveys underlying our microsimulation models imply that in Ghana 53% of household monetary expenditure is on goods and services that are, in principle, subject to VAT, while in Ethiopia the equivalent figure is 87%.

To a large extent this reflects the fact that the scope of exemptions varies significantly across countries. All six countries exempt certain goods and services: financial services; public services, including health and education services; water; and some foodstuffs. However, the range of foodstuffs that are exempt is relatively narrow in Ethiopia and Uzbekistan compared to the other four countries – only primary agricultural produce is exempt in Uzbekistan, for instance. Exemptions can extend to goods such as electricity (Ethiopia, Ghana, Senegal and Sri Lanka), transport services (Ghana, Sri Lanka, Uzbekistan and Zambia), oil and petroleum (Ghana), books and newspapers (Zambia) and textbooks (Ghana). In Ethiopia, Ghana, Sri Lanka and Zambia, firms with sales below a certain threshold are exempt from compulsory registration for VAT, while in Senegal firms of any size can, in principle, be required to register. Unusually, Uzbekistan employed a compulsory registration threshold based on the number of employees until 2019.

With the exception of exports, Ethiopia, Ghana, Senegal and Sri Lanka do not apply zero rates of VAT to any goods or services.^5^ Uzbekistan applies a zero rate to water and gas for household use, and Zambia applies a zero rate to a range of goods, including building supplies, mosquito nets, medical supplies, educational materials, energy saving equipment, and wheat flour and bread. Senegal has a reduced rate of 10% for accommodation and catering services owned by a licensed tourist accommodation provider.

It is clear that not all of these exemptions and reduced rates exist for equity reasons, or equity reasons alone. For instance, Zambia’s zero rate for energy saving equipment is likely there for environmental reasons (although as discussed in Ebrill et al. (2001), preferential VAT rates are typically a poor way of correcting for externalities, including environmental externalities). Senegal’s reduced rate of 10% for accommodation and catering services seems aimed at increasing the competitiveness of its tourism industry. Others, including those for financial services and small firms, likely reflect administration and compliance issues with VAT for these services.

Taken together, differences in tax rates and bases (as well as differences in tax compliance) contribute to significant differences in VAT revenues both as a proportion of GDP and as a proportion of overall tax revenues. For instance, Table 2.1 shows that VAT revenues were equivalent to 3.3% of GDP and 27% of tax revenue in Sri Lanka in 2017, reflecting a relatively narrow tax base compared to other countries in this study. In contrast, VAT revenues in Uzbekistan were equivalent to 6.9% of GDP and 39% of overall tax revenues in 2018 – reflecting a high tax rate and a relatively broad tax base. In general, VAT is a more important source of revenue in LMICs than in high-income countries: VAT and other general consumption taxes accounted for 21.2% of total tax revenues in OECD countries in 2018, for instance (OECD, 2020).

### 2.2 Cash transfer programmes

Each of the countries considered operates at least one non-contributory cash transfer scheme targeted at reducing poverty and supporting individuals and households deemed vulnerable. For the programmes we consider in Sri Lanka and Uzbekistan, eligibility is determined by household income but households must actively apply for the programmes themselves. In Ethiopia, Ghana, Senegal and Zambia, eligibility is based on a combination of geographic targeting to select which communities the scheme operates in, and proxy means tests and other defined criteria to assess which households in these communities should receive the transfer. In addition, in Ethiopia, Senegal, and Zambia, communities themselves help decide which households should receive a transfer: the aim is to improve targeting using local knowledge, but this can also lead to misallocation of transfers. Similar issues may arise through the administration of programmes: for instance, in Uzbekistan applications are processed by local Mahalla committees which need to verify informal and agricultural incomes that are difficult to observe, and ultimately have discretion in approving applications based on household composition and living conditions.

Ghana, Sri Lanka, Uzbekistan and Zambia’s schemes – Livelihood Empowerment Against Poverty (LEAP), Samurdhi, low-income family allowances, and the Social Cash Transfer Scheme (SCTS), respectively – are unconditional.^6^ Ethiopia’s Productive Safety Net Programme (PSNP) consists of a conditional transfer for households with able-bodied adults, who must take part in public work schemes, and an unconditional transfer for households where no adult is able to work. Senegal’s Programme National de Bourses de Sécurité Familiale (PNBSF) is a conditional cash transfer scheme which requires families to ensure their children are enrolled in school and properly vaccinated.

Recent years have seen significant expansions in some of the schemes. For instance, while there were 150,000 beneficiaries of Zambia’s Social Cash Transfer Scheme (SCTS) in 2015, the Government of Zambia extended coverage to close to 600,000 by 2017. Ethiopia’s PSNP has, since 2016, been expanding in to urban areas. However, the criteria used to determine eligibility can mean that significant fractions of poorer households are excluded from these programmes. For instance, Ghana’s LEAP requires that a household must contain someone who is either aged 65 or over, severely disabled, an orphaned or vulnerable child, or pregnant or with an infant – in addition to being extremely poor according to a proxy means test. Senegal’s PSBSF and the majority of Uzbekistan’s family allowances apply only to households with children. The geographical targeting can also mean that poor residents in certain parts of the country are not eligible for payments. Thus, even where these schemes are well targeted at poorer households, they do not necessarily provide a comprehensive social safety net for all households in or at risk of poverty. This is particularly true given the administrative challenge of implementing well-targeted programmes and possible caps on the total number of beneficiaries, which may mean that large numbers of eligible households are excluded in practice. These issues are important when considering whether these schemes represent viable alternatives to redistribution via the VAT system.

## 3 Modelling VAT and cash transfers

To model VAT and cash transfer systems and a hypothetical UT, we utilise household survey data, administrative revenue and expenditure data, input–output tables and microsimulation models.

### 3.1 Microsimulation models

We utilise bespoke microsimulation models for each country. ETHTAX and GHATAX (for Ethiopia and Ghana, respectively) were built by researchers at the Centre for Tax Analysis in Developing Countries (TaxDev); in each of the other four countries of study, the models used come from fiscal incidence models developed jointly by the World Bank and the Commitment to Equity Institute. Processed household survey datasets (see below) underlie each of these models, with detailed income and consumption information at the individual and household level, respectively, to which the rules of a country’s tax and transfer system are applied. In addition, each model embeds input–output tables to estimate how indirect taxes levied on production affect consumer prices, as described below.

These models are “static”, in the sense that they estimate the effect of tax and transfer policies holding fixed real quantities of goods consumed (and utilised in production processes) and labour supplied. This is a simplifying assumption (unless the goods and labour markets are characterised by perfectly inelastic demand and/or supply) but is in common with most of the preceding literature and applied practice studying the distributional effects of tax and transfer policies (e.g. Bourguignon and Pereira da Silva, 2003, Bourguignon and Spadaro, 2006, Del Granado et al., 2012, Lustig, 2018).

Incorporating behaviour would require estimating or assuming both demand and supply elasticities for many different goods and services, as well as labour. This is difficult to do convincingly without plausibly exogenous variation in the prices and wages households face, and in the absence of pre-existing estimates for the requisite elasticities in the countries we study. In addition, whereas estimating behavioural elasticities typically requires collapsing the consumption data reported in household surveys into a small number of categories to ensure tractability, our static approach allows us to use very detailed consumption data in our modelling. This allows us to better account for *existing* heterogeneity in consumption and labour supply choices across households.

All our models measure real income as consumption per capita, inclusive of home-produced and bartered goods, and net of indirect taxes and subsidies (“consumable income”).^7^ This allows us to capture the impact of both transfers and indirect taxes on households in a simple framework. Because we hold behaviour fixed, our estimates of the impact of policy changes on real income represent first-order approximations of the true welfare effects of the policies: to the extent that households and individuals change their behaviour in response to the reforms, the welfare gain (cost) of a net tax reduction (increase) would be higher (smaller). By holding behaviour fixed, our static modelling approach will also in many cases somewhat overestimate the revenue costs (yields) from net tax cuts (increases). This is because of income effects – the higher (lower) real income result from net tax cuts (increases) would typically lead to increases (reductions) in expenditure, generating increases (reductions) in tax revenues that would partially offset the direct mechanical effects of the reforms. However, this will not always be true: if substitution effects are large and work in the opposite direction to income effects, the true revenue costs (yields) from net tax cuts (increases) can be underestimated. Fully general statements about the potential biases resulting from our static modelling approach would therefore require estimating or assuming the behavioural elasticities that the approach is designed to avoid the need for.

### 3.2 A cost-push model of VAT incidence

In common with most applied analyses of the distributional effects of taxes and transfers, we assume that VAT is pushed forward on to the prices paid by purchasers of products, and is hence ultimately economically incident on consumers. This would be consistent with either perfectly elastic supply or completely inelastic demand for each product if markets are perfectly competitive or characterised by imperfect competition with fixed mark-ups.

It is unlikely that all product markets can be characterised in this way. However, studies suggest that consumers typically bear a large proportion of the incidence of VAT – particularly for increases in VAT, which are the focus of this paper (Benzarti et al., 2020, Carbonnier, 2007, Gaarder, 2019, Lyssiotou and Savva, 2021). Moreover, the assumption of full incidence on consumers significantly simplifies the modelling of the distributional effects of VAT. In particular, for a perfectly functioning VAT system without exemptions, it is possible to estimate household VAT payments using only data on expenditures. In contrast, if the incidence of VAT were partly (or fully) on capital, land or labour, then detailed information on the production side of the economy – including not only production chains, but also who owns what capital and land, and who works where – would be required.^8^

When there are VAT exemptions, an assumption of full incidence on consumers still avoids the need for information on capital, land and labour, but in addition to information on household expenditures, information on production chains is required for modelling VAT. This is because a VAT exemption means that while sellers are not required to charge VAT on their output, neither are they able to reclaim any VAT paid on inputs to their production process. Under cost-push assumptions, this input VAT becomes embedded in the price of exempt goods and services. In other words, the purchaser of an exempt product may still face the burden of VAT on the inputs used in producing that product. If that purchaser is another firm, then that embedded VAT would be reflected in the prices they must charge, on top of any VAT levied directly on their own output. This means the purchaser of a VATable (or zero-rated) product may face additional embedded VAT on top of the statutory rate of VAT on the product in question.

To account for these effects, we use input–output tables, and build upon a method developed by Ahmad and Stern (1984) and elaborated by Coady (2008).^9^ In particular, we assume that products are produced according to Leontief production technologies characterised by the intermediate input usage reported in input–output tables. This means that we assume that inputs are used in fixed proportions, and the elasticity of substitution between inputs is zero.

Normalising prices in the absence of VAT to 1 for each of N products, the vector of *effective* VAT rates for domestically produced varieties of these N products, ***T****,* that incorporates this embedded VAT can be characterised by:

(1) $$TX=tX+T\Gamma^{D}X-\left(E\circ t\Gamma^{D}\right)X+t\Gamma^{F}X-\left(E\circ t\Gamma^{F}\right)X$$

• T is the 1 × N vector of effective VAT rates.
• X is a diagonal N × N matrix of domestic outputs of these products.
• t is a 1 × N vector of statutory VAT rates applicable on the sales of each sector.
• $\Gamma^{D}$ and $\Gamma^{F}$ are N × N matrices of technical coefficients for domestic and imported inputs for each sector, respectively. Each coefficient *i,j* in these matrices captures the share of the cost of product *j* that is made up of the cost of each product *i* ∈ N as an input into the production of product j, separately for domestically produced inputs (in matrix $\Gamma^{D}$) and imported inputs (in matrix $\Gamma^{F}$).
• E is a 1 × N vector where each entry is equal to 0 if the corresponding sector cannot reclaim VAT paid on inputs (e.g. it is VAT exempt), and is equal to 1 otherwise.
• ° is the Hadamard product.

The effective VAT rate ($T_{i})$ on product *i*, is a function of the statutory VAT rate ($t_{i}$) on that product, the inputs used in its production (summarised by the technical coefficient matrices $\Gamma^{D}$ and $\Gamma^{F}$), the effective VAT rates on those inputs (T), the statutory VAT rates on those inputs (t) and whether the producers of product *i* can reclaim the statutory VAT rates levied on their inputs (recorded in vector E).

The key reason we distinguish between domestic inputs ($\Gamma^{D}$) and imported inputs ($\Gamma^{F}$) is that, abstracting from the use of domestic inputs in the production of imported inputs (e.g. due to global value chains), the price of imported inputs should not be directly affected by unrecoverable domestic input VAT, only the statutory rate of VAT (t), in a pure cost-push model. This is in contrast to the price of domestic inputs, which can be affected by unrecoverable domestic input VAT, such that their price is affected by the overall effective rate of VAT (T). Of course, if imports and domestic inputs are substitutable in production and consumption, the cost-push assumption may not hold for tradable goods: rather than being pushed forward via prices, unrecoverable input VAT would be at least partially borne by factors of production (capital, land, labour). We maintain the cost-push assumption, however, so that we can model the distributional effect of all unrecoverable input VAT (on consumers); modelling the impact of any input VAT pushed back to factors of production would require additional data not typically available in household surveys. One way to rationalise this would be if imports and domestic varieties of products were different inputs in the Leontief production functions our cost-push framework is based on.

In principle, as well as being used to account for VAT exemptions that prevent producers of particular products being unable to reclaim input VAT, vector E could be used to account for small and informal/tax-evading firms, which are an important feature of LMICs. This would require the N products in our input–output matrix to include two varieties of each product: one for large and formal/tax-compliant firms, for which $E_{i}=1$ unless the product itself were exempt; and another for small and informal/tax-evading firms, for which $E_{i}=0$. The statutory tax rate for these two varieties would be the statutory tax rate on output for the product in question, and 0, respectively, reflecting the fact that the small and informal/tax-evading firms would not charge VAT on their outputs.^10^

However, input–output tables generally do not contain separate entries for large/tax-compliant and small/tax-evading firms selling the same product. This means if one wanted to utilise this approach, one would need to partition existing product categories into varieties produced by large/tax-compliant and small/tax-evading firms. Both the importance of these two types of firms to overall industry output, and the use of inputs from these two types of firms, is likely to differ by product category (and in the case of input usage, by type of firm (De Paula and Scheinkman, 2010)). However, information on both outputs and input-usage by firm type and product category is not available for our countries. Nor is information on place of purchase by consumers in our main survey datasets, which can be used as a proxy for whether consumers are purchasing from the informal and formal sector. We are not aware of a context where all of the requisite data noted here exists in tandem, but pursuing this line of analysis would be a useful methodological extension to the current research if and when such data is available.

In the absence of such information, our main results therefore assume that the prevalence of small/tax-evading firms is constant across product categories for domestically produced products and that the share of purchases from such traders is constant across the population. The scale and direction of any biases this generates in our results will depend on (a) how the share of purchases from small/tax-evading firms varies across households, and (b) how the prevalence of small/tax-evading firms varies across different goods and services markets. In relation to (a), Bachas, Gadenne and Jensen (2021) find that in countries where household survey data include proxies for the size and tax status of the firms that consumers purchase from, poorer households are consistently more likely to purchase from small/tax-evading firms. In this case, our assumption will mean that we are likely to *overestimate* the progressivity of preferential VAT rates. This is because a smaller-than-average fraction of poorer households’ expenditure would be subject to the extra VAT due if preferential VAT rates were abolished (given more of their expenditure is with small/tax-evading firms that would still not pay VAT). In relation to (b), Bachas, Gadenne and Jensen (2021) find that food products are more likely to be purchased from small/tax-evading firms. Because food products represent a large share of expenditures subject to preferential VAT rates in the countries we study, this means we likely overestimate the cost of preferential VAT rates. This is because small/tax-evading firms still would not pay VAT if preferential VAT rates were abolished. In general, we would therefore expect preferential VAT rates to be even less progressive, but smaller in scale relative to existing VAT revenues, than suggested in our baseline results reported in Section 4. To explore this, Appendix C presents additional results for Senegal, imputing data from a separate household survey which does contain proxies for firm size and tax status of establishments where purchases are made, allowing us to relax our baseline assumptions.

We also assume the full statutory rate of VAT due on a product is collected on imports. This reflects the fact that VAT compliance for imports is likely to be significantly higher than for domestic production, because VAT is collected as part of customs clearance, and exemptions for small firms do not apply to imports (irrespective of the size of the exporting firm overseas).

Let α be a scalar equal to the share of domestic output produced by small/tax-evading firms. In this instance, equation (1) becomes:

(2) $$TX=\left(1-\alpha \right)tX+T\Gamma^{D}X-\left(1-\alpha \right)\left(E\circ t\Gamma^{D}\right)X+t\Gamma^{F}X-\left(E\circ t\Gamma^{F}\right)X$$

Rearranging this equation to solve for T, we obtain:

(3) $$T=\left(\left(1-\alpha \right)t-\left(\left(1-\alpha \right)E\circ \left(t\Gamma^{D}\right)\right)+t\Gamma^{F}-\left(E\circ \left(t\Gamma^{F}\right)\right)\right){\left(I_{N}-\Gamma^{D}\right)}^{-1}$$

Equation (3) tells us that the effective tax rate $\left(T_{i}\right)$ on the domestic variety of a product is higher when: the statutory tax rate ($t_{i})$ is higher; and the share of output produced by small/tax-evading firms (α) is lower.^11^ The impact of the use of domestic ($\Gamma^{D}$) versus foreign inputs ($\Gamma^{F}$) is ambiguous. On the one hand, domestic inputs may have embedded VAT on them while imports cannot, such that all else equal, greater usage of domestic inputs can increase the effective tax rate. However, a proportion of domestic inputs may be from small/tax-evading firms which do not charge (and remit) VAT on their outputs. This implies that greater usage of domestic inputs relative to imported inputs may reduce the effective tax rate. The overall effect of the balance between domestic and imported inputs therefore depends on the relative scale of unrecoverable input VAT (T-t) and the prevalence of small/tax-evading firms (α).

We model the overall vector of effective tax rates faced by consumers (τ) as a weighted average of the vector of effective tax rates on domestically produced outputs (T), the statutory tax rates on imports (t), and the shares of each product that are imported (**β**). Defining $1_{N}$ as the N row-vector of ones:

(4) $$\tau =\left(1_{N}-\beta \right)\circ T+\beta \circ t$$

In order to implement this method, in each country we use input–output tables or social accounting matrices (SAMs), and calibrate key parameters (α**, *β****,* $\Gamma^{D}$and $\Gamma^{F}):$

• In each of our six countries, the original categorisation of products in the input–output table or SAM is not detailed enough to separately identify all products subject to different VAT treatments (e.g. zero rates, standard rates and exemptions). In this instance we partition the original product categories into sub-categories subject to different VAT treatments, ensuring that the original input–output accounting identities remain intact.^12^ In general, we partition according to the share of each sub-category in the overall category as reported in our household expenditure data, although in a few instances where there is evidence that firms and consumers make use of sub-categories in significantly different proportions we deviate from this approach (instead using estimates from other data).^13^ Each sector of our partitioned matrices is then manually matched to the statutory VAT rules of the country for our year of analysis.
• We calibrate α using official revenue data and our microsimulation models. First, we multiply actual VAT revenues reported by each country’s tax authority by the share of national consumption captured in the household survey (see Table 3.1). This yields a revenue target; we then iteratively re-estimate VAT revenues under status quo tax systems in each country, adjusting α until the estimate matches the target.

**Table 3.1:** Overview of data used in each country.

**Country**	**Household survey(s)**	**Survey year**	**Consumption total**	**SAM/I-O year**
Ethiopia	Ethiopian Socioeconomic Survey and Household Consumption and Expenditure Survey	2015–16	88%	2005–06
Ghana	Ghana Living Standards Survey	2012–13	105%	2005
Senegal	Enquête de Suivi de la Pauvreté au Sénégal	2011	64%	2011
Sri Lanka	Household Consumption and Expenditure Survey	2016	44%	2011
Uzbekistan	Listening to the Citizens of Uzbekistan Household’s Survey	2018	73%	2015
Zambia	Living Conditions Monitoring Survey	2015	44%	2007

Note: Coverage of survey is defined as the ratio of aggregate household consumption as measured in the household survey to final national household and NPISH sector consumption as estimated in national accounts. For Ethiopia, information from the consumption survey is imputed into the socioeconomic survey based on observable household characteristics due to a lack of detail on expenditure in the latter. More details are available from the authors on request.
• ***β*** is the share of each product that is imported, accounting for shipping costs and import duties (which form part of the VAT base), which we take from each country’s SAM or national accounts.
• We partition the overall matrix of Leontief coefficients (Γ) into the matrices of domestic ($\Gamma^{D}$) and imported ($\Gamma^{F}$) coefficients, under the assumption that each product *i* using product *j* as an input makes use of domestically-produced and imported varieties of project *j* in the same proportion as the overall economy – which is captured by ***β***. This is because the input–output tables and SAMs for our countries do not distinguish between the use of domestically-produced and imported varieties of products (∀*j* ∈ N) as inputs into the production of products (∀*i* ∈ N), or alternatively distinguish between domestic inputs and imported inputs but not the product category of imported inputs.^14^

With more detailed information on input usage and the prevalence of small/tax-evading firms by sector we would not need to make these additional assumptions, but they are necessary given the data available in our six countries, which are typical of most LMICs.

### 3.3 Modelling cash transfers

In addition to modelling indirect taxes, our microsimulation models allow us to simulate households’ cash transfer entitlements under existing and hypothetical transfer systems in four out of six countries. These simulations are based on household socio-economic data from the underlying household surveys and the eligibility rules and payment rates for each cash transfer. For instance, in Ghana and Senegal we combine estimated PMT scores with demographic and geographic targeting, respectively. In Sri Lanka and Zambia, payments are simulated based on household characteristics according to programme eligibility criteria; in the former case we do so for households who report receiving the benefit at all, thus combining both self-reports with simulations. We adopt this approach partly due to the recent rapid scale up of cash transfer programmes in many countries; reported receipts in household survey data that is a number of years old would underestimate entitlement to existing cash transfer programmes. In Ethiopia and Uzbekistan, however, the household survey data does not include the requisite information to model the main cash transfer programmes – the PSNP and family allowances, respectively – and we therefore use reported benefit receipt observed in the data instead of simulating entitlement. As the data used in these two countries comes from the same year as the policies we model, scale-up of programmes in the intervening years is not a concern, but note that because the survey data for Ethiopia was collected before the roll out of the PSNP to urban areas, our estimates include the rural PSNP only.

We note that both simulated entitlements and self-reported receipts carry potential biases. Using simulated values provides a useful picture of the targeting of a programme in principle but might not accurately reflect actual disbursements. It is well known that cash transfer programmes are subject to inclusion and exclusion errors, and simulations impose that these errors are zero. Furthermore, using simulated values typically assumes full take-up of benefits by those who are eligible to receive them, which is unlikely to be true in reality. Benefit receipts in surveys, on the other hand, can be subject to significant misreporting which may be correlated with other characteristics (Mittag, 2019, Bruckmeier et al., 2021).

### 3.4 Data

The main sources of data for our analysis are household surveys, which ask a representative sample (once weighted) of households about their incomes, expenditures and other socio-economic characteristics (Table 3.1). In order to use these datasets for more recent policy years (see Table 2.1), we take account of changes in aggregate household consumption as recorded in national accounts but must assume that the broad composition of consumption does not change significantly over the space of a few years.

In two of our six countries (Ethiopia and Ghana), the (weighted) aggregate value of consumption reported by households is fairly close to the aggregate value of household consumption reported in national accounts. In the other four countries (Senegal, Sri Lanka, Uzbekistan and Zambia) the household surveys significantly under-record household consumption relative to national accounts. This under-recording means that our microsimulation models will also underestimate both VAT revenues and the cost of reduced VAT rates in cash terms. However, given that this reflects underestimates of consumption, estimates of VAT payments and the cost of preferential VAT rates as a percentage of consumption should be significantly more accurate.

In addition to household survey data and national accounts data, we utilise input–output (I-O) tables and social accounting matrices (SAMs) to model unrecoverable VAT paid on intermediate inputs into the production of consumer products. Table 3.1 shows that these data are typically older than the household survey data: while patterns of intermediate input usage may have changed somewhat in the intervening years, this is unlikely to influence results in a significant way. A full list of data used in each country is provided in Appendix B.

## 4 The impact of preferential VAT rates

In this section we analyse those preferential VAT rates that are in place for reasons other than administrative efficacy. To do this, we simulate a policy counterfactual where there are no reduced rates or exemptions except for those on public, health, education and financial services, domestic rents and small traders.^15^ We then estimate the ‘tax expenditure’ associated with these non-administrative preferential VAT rates for each household, presenting mean impacts by decile groups of the population consumption distribution, as well as estimated effects on summary poverty and inequality measures.

It is worth reiterating a couple of implications of our approach to modelling VAT as set out in Section 3. First, our models make simplifying assumptions about tax incidence and behaviour. We assume that the full incidence of a broader VAT base is borne by consumers in the form of higher prices. Whether alternative incidence assumptions would suggest that existing preferential VAT rates are more or less progressive depends on the distribution of labour and capital incomes derived from formal sector firms who would be affected by the broader VAT. While we cannot account for such income flows with the data available, evidence from similar work in Mexico has suggested that alternative incidence assumptions may make VAT increases more progressive (Abramovksy et al., 2011). Our assumption of no behavioural response suggests that our estimates of VAT expenditures constitute upper bounds on their true revenue effects. This is because if the preferential VAT rates were abolished, household real incomes would be lower, likely leading them to reduce overall expenditure, in turn reducing tax revenues, offsetting part of the mechanical increase in revenue resulting from the preferential VAT rates. Moreover, substitution effects would go in the same direction in this instance.^16^ Second, our assumption that the share of purchases that are from small/tax-evading firms is constant across products and households means that it is more likely that in general we are overestimating rather than underestimating both the progressivity and the cost of preferential VAT rates. The additional results included in Appendix C for Senegal suggest that our baseline results for that country are likely to overstate the cost of preferential rates of VAT but not necessarily the progressivity.

**Figure 4.1:**
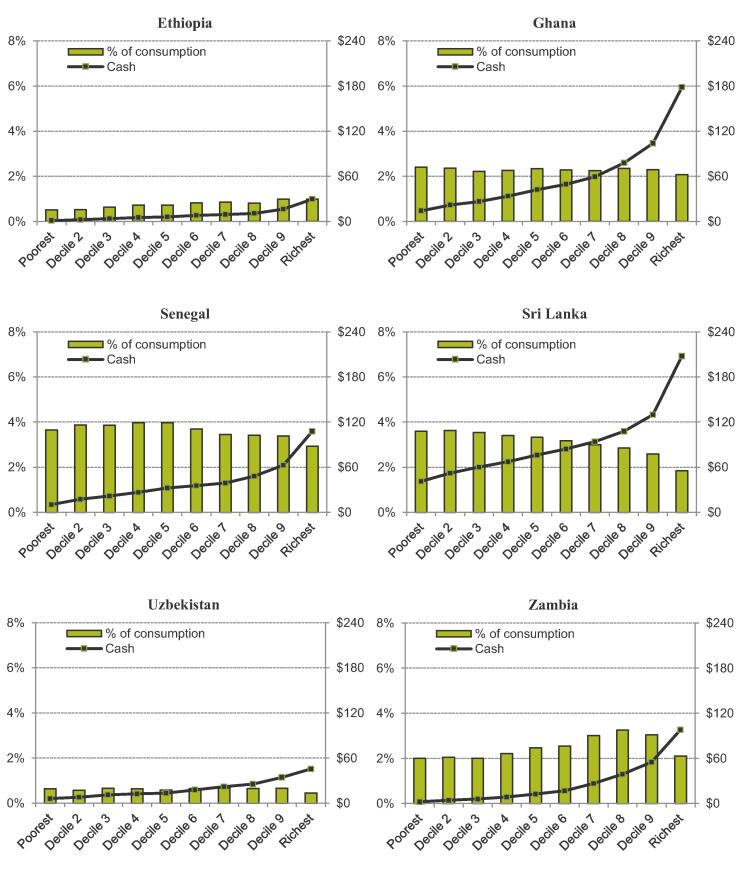
Tax expenditure on preferential VAT rates across the consumption distribution in six LMICs *Note:* Population decile groups ranked by per capita consumption; cash amounts are annual USD 2011 PPP. *Source:* Author’s calculations using GHATAX, ETHTAX and CEQ/World Bank fiscal incidence analysis.

Notwithstanding these caveats, Figure 4.1 shows estimated VAT expenditures by country and consumption decile, and suggests a mixed picture with regards to their progressivity. In Sri Lanka, preferential VAT rates are progressive overall when assessed as a proportion of consumption; Senegal also exhibits a somewhat progressive structure, though not strongly so. However, in Ghana and Uzbekistan the trend is less clear across the consumption distribution and in Ethiopia and Zambia preferential VAT rates actually appear to be slightly regressive. This is partly driven by considerable heterogeneity in the relative contribution to total consumption of monetary expenditure compared to home production and barter across the consumption distribution. In particular, the poorest decile groups attribute more of total consumption to home production and barter – which is untaxed – than monetary expenditure. This means that exemptions directly benefit the poorer decile groups less.

When one considers the per capita benefit received from preferential rates in cash terms the pattern is consistent across the six countries. Even when poorer households benefit more in proportional terms, the fact that richer households spend significantly more on the food products and other goods subject to preferential rates of VAT in absolute terms means that they obtain a much larger implicit cash subsidy from the preferential rates. Taking the case of Sri Lanka – where existing exemptions are most clearly progressive in relative terms – an individual in the poorest decile group of the population can, on average, attribute 3.6% of the value of their consumption to preferential VAT rates on the goods that they buy, compared to 1.8% for those in the top decile group. However, the average estimated annual benefit received in cash terms is $41 per capita in the lowest decile group compared to $208 per capita at the top of the distribution. This pattern suggests that even if preferential VAT rates can be described as progressive in some cases, they are not “pro-poor”.^17^.

Despite the fact that richer individuals benefit substantially from preferential VAT rates, it is important to note that, considered in isolation, they do reduce the incidence of poverty by increasing the real spending power of the population. Table 4.1 indicates the estimated marginal effects on poverty of preferential VAT rates at the poverty lines defined by the World Bank for Low Income, Lower-Middle Income and Upper-Middle Income countries.^18^ Results are displayed for the poverty headcount ratio (which measures the proportion of the population that falls below a poverty line) and the poverty gap index (which in this case measures the proportion of national consumption that would be required to lift all of those below the poverty line out of poverty).^19^ Once again, the size of these effects differs greatly depending on the total size of the exemptions granted, but they can be substantial. In Senegal, the poverty headcount ratio is estimated to be reduced by 2.90 percentage points at the lowest poverty line as a result of preferential VAT rates under our baseline modelling assumptions.^20^ After incorporating informality in Appendix C, this effect is reduced to 2.68 percentage points, highlighting that the effect sizes in Table 4.1 are likely to be upper bounds.

**Table 4.1:** Estimates of the marginal effects on poverty from current preferential VAT exemptions rates.

**Poverty line**	**$1.90 per day**		**$3.20 per day**		**$5.50 per day**	
**Measure**	**Headcount**	**Gap**	**Headcount**	**Gap**	**Headcount**	**Gap**
Ethiopia	–0.29	–0.14	–1.08	–0.34	–0.45	–0.35
Ghana	–0.68	–0.21	–1.20	–0.53	–1.48	–0.91
Senegal	–2.90	–1.26	–1.29	–1.65	–0.69	–1.48
Sri Lanka	–0.40	–0.08	–1.97	–0.46	–2.37	–1.24
Uzbekistan	–0.05	–0.01	–0.43	–0.09	–0.41	–0.23
Zambia	–0.87	–0.69	–0.91	–0.86	–0.60	–0.81

Note: All figures are percentage point changes in poverty rates or gaps. Poverty figures are calculated based on consumable income per capita pre- and post-reform.

Source: Author’s calculations using GHATAX, ETHTAX and CEQ/World Bank fiscal incidence analysis.

A second conclusion from the results in Figure 4.1 is that the scale of the tax expenditure provided in the form of preferential VAT rates varies significantly across the sample. In Uzbekistan, their value is estimated at 0.6% of consumption on average whereas in Senegal this figure is 3.8%. This implies significant variation in the potential revenue foregone and the opportunity to redistribute more effectively through other means. This is driven by a combination of factors: Senegal grants exemptions and reduced rates to a wider range of goods and services, it has no minimum turnover threshold and thus all firms are supposed to register for VAT in principle, and its market consumption as a share of overall consumption is high. Uzbekistan, on the other hand, has few preferential VAT rates; such factors drive variation across the countries in our sample.

Table 4.2 provides estimates of the revenue foregone through the provision of preferential VAT rates. Exemptions and reduced rates are generally costly, with estimates of up to half of baseline VAT revenues in Sri Lanka (where exemptions are broad). Uzbekistan stands out here with a relatively narrow range of preferential VAT rates (aside those which are likely administratively motivated) and therefore much more limited revenue potential. Again, these are upper bound figures due to both behavioural response and patterns of informality; in the latter case, additional analysis in Appendix C suggests that greater informality in currently sectors receiving preferential rates reduces estimated VAT expenditures from over a third of baseline VAT revenues to 26% in Senegal.

**Table 4.2:** Estimated revenue cost of preferential VAT rates in six LMICs.

**Country**	**% of VAT revenue**	**% of tax revenue**	**% of GDP**
Ethiopia	16.1%	5.0%	0.6%
Ghana	32.5%	9.1%	1.0%
Senegal	33.7%	12.1%	2.0%
Sri Lanka	49.8%	13.2%	1.7%
Uzbekistan	2.6%	1.0%	0.2%
Zambia	37.9%	11.8%	1.7%

Note: All figures in this table are adjusted to account for the under-recording of aggregate consumption in the household survey datasets used.

Source: Author’s calculations using GHATAX, ETHTAX and CEQ/World Bank fiscal incidence analysis. Revenue data comes from national sources and CPI inflation and PPP conversion figures come from the World Bank.

## 5 The impact of cash transfers

Preferential VAT rates are not well targeted towards poor households overall. However, we must also consider whether there are better alternative policy options for redistribution. As discussed in Section 2, each of our six sample countries have either a conditional or an unconditional cash transfer scheme. These vary in scale and the specific groups of the population that they target but all have in common that they aim to reach some of the poorest and most vulnerable members of society. In this section, we present estimates of cash transfer receipts for households across the consumption distribution in each country, focusing on the main programme aimed at poor households in each case.

**Figure 5.1:**
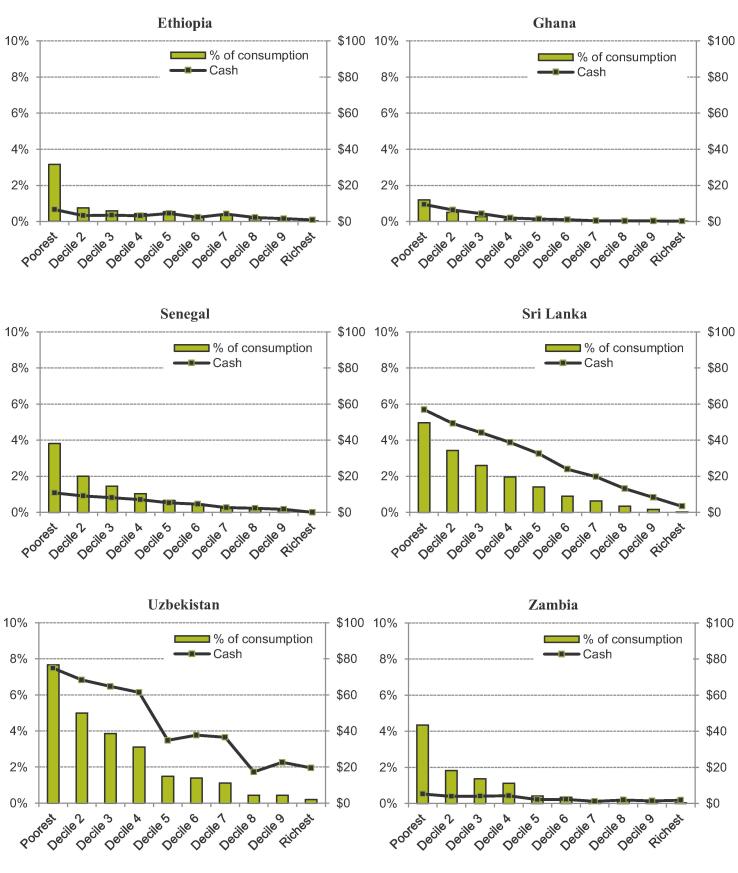
Cash transfer payment receipts across the consumption distribution in six LMICs *Note:* Population deciles ranked by per capita consumption; cash amounts are annual USD 2011 PPP. Estimates for Ethiopia and Uzbekistan based on reported transfer receipts. Estimates for other countries based on simulated transfer receipts. *Source:* Author’s calculations using GHATAX, ETHTAX and CEQ/World Bank fiscal incidence analysis.

Figure 5.1 plots the distributional impact of existing cash transfer schemes in each consumption decile group. As described in Section 3.B, estimates of the transfer amount received by each household come from simulated values (Ghana, Senegal and Zambia), receipts observed in the survey data (Ethiopia, Uzbekistan), or a combination of both (Sri Lanka). For each per-capita consumption decile, Figure 5.1 shows cash transfer receipts in both per capita terms and as a share of total consumption, averaged across both those who do and do not receive the transfers. The results indicate that on the whole these cash transfer schemes are fairly well targeted and have a positive redistributive effect both relatively and in absolute terms – i.e. they are both progressive and pro-poor. However, targeting is imperfect, with some evidence of “leakage” of transfers into higher deciles – average cash disbursements for Ethiopia’s PSNP programme change little between the second and eighth consumption deciles, for instance. These conclusions hold both in countries where receipts reported by the household are used and in cases where entitlements are simulated.

**Figure 5.2:**
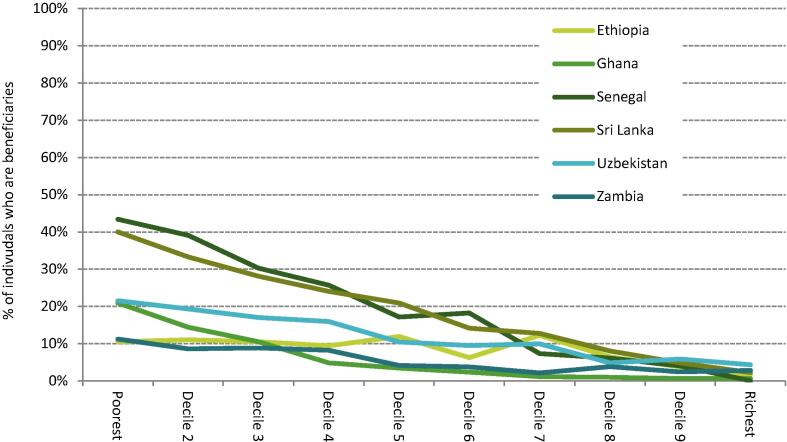
Coverage rates of existing cash transfer programmes across the consumption distribution in six LMICs. *Note:* Population deciles ranked by per capita consumption; an individual is counted as covered by a cash transfer scheme if any member of their household receives a positive cash amount in that year. *Source:* Author’s calculations using GHATAX, ETHTAX and CEQ/World Bank fiscal incidence analysis.

In addition, although on average these schemes appear to be fairly well targeted, a decile chart of averages masks heterogeneity within deciles. Figure 5.2 displays estimated coverage rates for each decile group. In the bottom consumption decile, for example, the highest coverage rate achieved is 43% in Senegal; in Ethiopia and Zambia this falls to 11%. While there may be a degree of misreporting in self-reported receipts when used, these patterns are likely to more substantively reflect that these schemes tend to focus on subsets of the population, as highlighted in Section 2. They are often provided for particular demographic groups or regions and thus miss large numbers of households at the bottom of the consumption distribution, even before considering potential exclusion errors in their targeting.

A final conclusion from these results is that in most cases the scale of the benefits disbursed through these cash transfer programmes is dwarfed by VAT expenditures, with possible implications for the feasibility of transfer programme scale-up. The exceptions in this respect are the two highest-income countries in our sample – Sri Lanka and Uzbekistan. In both these countries, the cash transfer schemes we analyse are comparatively large in terms of beneficiaries and payment amounts, which may reflect the greater (administrative and fiscal) feasibility of broader social safety nets as countries develop.

## 6 Compensating for VAT base broadening with a UT

Taken together, the results from 4, 5 show that preferential VAT rates in the six countries studied are poverty-reducing but benefit richer households the most in cash terms, and that while existing cash transfer programmes are more targeted at poor households, they are sometimes small-scale, and reach only a relatively small proportion of poor households. This means that if the six countries studied were to abolish their preferential VAT rates, significant reform and expansion of existing programmes would be required if governments wished to use the proceeds to tackle poverty – not just in terms of their generosity, but also in terms of population coverage too. While additional tax revenues would provide many options for governments to support poor households, including reductions in other taxes or expansion of public services, such policy options are difficult to compare across countries.

We therefore consider a hypothetical uniform per capita cash transfer scheme, which allows us to contrast the redistributive impact of a counterfactual policy option in a simple and comparable way in each country. Such programmes – known as Universal Transfer (UT) schemes or, more recently, Universal Basic Incomes (UBI) – are rare in practice but have received increasing political attention in recent years.^21^ Evaluations are underway or completed in Kenya, India and Namibia, for instance, but the biggest scheme so far is Iran’s UT, which was introduced in 2010 to replace energy price subsidies: the per-capita payments were equivalent to 28% of median disposable income per capita, and cost 6.5% of GDP (Salehi-Isfahani and Mostafavi-Dehzooei, 2018).^22^ While our countries of study do not currently have equivalent programmes in place, the example of Iran – where bank accounts were set up for more than 60 million people – suggests such schemes may be increasingly feasible. We return to important broader questions about work incentives and programme administration later; for now, we abstract from such considerations and focus on the possible redistributive effects of a policy that is completely untargeted, in comparison to existing tax expenditures via the VAT system.

**Figure 6.1:**
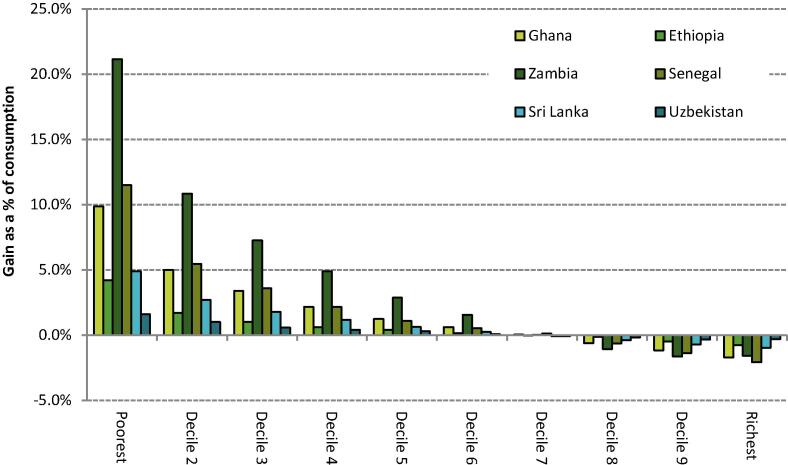
The distributional impact of a implementing a uniform VAT and using 100% of the revenue gain to fund a UT in six LMICs *Note:* Population deciles ranked by per capita consumption; UT is funded from the full revenue gain from implementing a uniform VAT at the standard rate in each country (excluding public services, financial services and accommodation costs) and is distributed per capita. *Source:* Author’s calculations using GHATAX, ETHTAX and CEQ/World Bank fiscal incidence analysis.

For each country we simulate the same abolition of preferential VAT rates as in Section 4 but now simultaneously introduce a UT funded by the revenue yield from broadening the VAT base. Figure 6.1 plots the estimated average distributional impact of such a reform, using 100% of the extra VAT revenue to fund the UT. It is clear that such a reform would benefit the poorest deciles of the consumption distribution, on average. Indeed, it is trivial that a UT will be more redistributive than a benefit that increases in absolute terms with overall consumption, such as the preferential VAT rates in the countries we study. Preferential VAT rates would only be more progressive than a UT if poor households spent more in cash-terms on goods or services subject to the preferential rates than rich households.

Figure 6.1 highlights non-trivial differences in the magnitude of possible redistribution from this reform by country though: contrast the 1.6% gain in consumption value for the bottom decile in Uzbekistan with the 18.5% gain in Zambia. Fundamentally, these differences are driven by three factors: the overall cost of existing preferential VAT rates (which determines the size of the UT that can be funded); the degree of relative progressivity of these preferential rates (which determines the relative loss from a broader VAT base), and overall consumption inequality (which determines the relative impact of the UT). The latter drives a substantially larger percentage gain for poor households in Zambia compared to Ghana, despite existing preferential VAT rates having a similar scale and similar degree of relative progressivity in these countries (see Figure 4.1) – Zambia is one of the most unequal countries in the world.

Individuals towards the top of the distribution would lose out from the modelled reform overall because although they receive the same UT payment, the cash-terms benefit they currently enjoy as a result of preferential VAT rates but would lose under the reform is much bigger than for poorer households. Figure 6.1 shows that under the modelled revenue-neutral scenario, households in the bottom six deciles of the consumption distribution would be fully compensated in each of the countries studied, on average. In principle though, the reform could be made revenue-raising while still more than compensating poor households by reducing the UT somewhat. The scope for revenue-raising this way again depends on not only on the relative progressivity of existing VAT expenditures but also the degree of consumption inequality. For instance, introducing a UT that, on average, fully compensated the bottom three deciles of the consumption distribution for the abolition of preferential VAT rates would require 44% of the additional revenue raised from a broader VAT base in Ghana, and only 22% in Zambia – with these differences again reflecting Zambia’s higher levels of inequality.

Given that the reform would redistribute resources from richer to poorer households, it would seem to have the potential to be both poverty- and inequality-reducing. The bottom panel of Table 6.1 provides estimates of the marginal effects of the modelled reform on the poverty headcount and poverty gap measures at three different international poverty lines. The top panel of the table shows the estimated impact of the cash transfer schemes studied in Section 5 on these same metrics, providing a benchmark for comparison with our modelled UT reform. However, it is important to note that these estimates are not directly comparable because the poverty impacts of the VAT and UT reform are for a revenue-neutral reform rather than programmes with a net fiscal cost, and would come on top of the impacts of existing cash transfers.

**Table 6.1:** Estimates of the marginal effect on poverty of UT funded by abolition of preferential VAT rates.

**Poverty measure**	**$1.90 per day**		**$3.20 per day**		**$5.50 per day**	
**Existing cash transfer**	**Headcount**	**Gap**	**Headcount**	**Gap**	**Headcount**	**Gap**
Ethiopia	–0.26	–0.21	–0.19	–0.23	–0.00	–0.15
Ghana	–0.15	–0.09	–0.10	–0.11	–0.08	–0.08
Senegal	–0.71	–0.53	–0.05	–0.41	–0.00	–0.26
Sri Lanka	–0.76	–0.21	–1.75	–0.69	–0.94	–1.01
Uzbekistan	–1.91	–0.86	–2.63	–1.46	–1.11	–1.55
Zambia	–0.11	–0.31	–0.01	–0.21	–0.01	–0.13

**Additional effect of UT**	**Headcount**	**Gap**	**Headcount**	**Gap**	**Headcount**	**Gap**

Ethiopia	–0.48	–0.27	+0.05	–0.17	+0.13	–0.03
Ghana	–1.10	–0.45	–1.15	–0.73	+0.12	–0.63
Senegal	–0.80	–1.23	–0.06	–0.83	+0.22	–0.33
Sri Lanka	–0.53	–0.09	–1.27	–0.47	–0.51	–0.74
Uzbekistan	–0.13	–0.04	–0.22	–0.15	–0.23	–0.21
Zambia	–0.53	–1.46	+0.52	–0.82	+0.34	–0.32

Note: All figures are percentage point changes, with Headcount referring to the poverty headcount and Gap referring to the poverty gap. Poverty figures are calculated based on consumable income per capita pre- and post-reform using three international poverty lines in PPP USD. Existing cash transfer shows impact on each poverty metric of the cash transfer programme described in Section 2.B. Additional effect of UT shows the additional impact on each poverty metric of broadening the VAT base and introducing a revenue-neutral universal cash transfer.

Source: Author’s calculations using GHATAX, ETHTAX and CEQ/World Bank fiscal incidence analysis.

The bottom panel shows that the modelled VAT and UT reform would reduce poverty headcounts at the lowest poverty line in all cases. These effects are potentially large: for Ghana there is a reduction in poverty of 1.1 percentage points, or over 310,000 individuals; for Ethiopia the corresponding figures are 0.5 percentage points or almost 600,000 individuals. Different baseline poverty rates mean that percentage point changes have different interpretations depending on the context, however. For instance, while we estimate a similar percentage point impact in both Ethiopia and Sri Lanka, the much higher rates of poverty in the former implies that the size of the population in extreme poverty is reduced by less than 1.5%, compared to well over a third in Sri Lanka. A small percentage point reduction in Uzbekistan contributes to a 6% reduction in the size of the population in extreme poverty.^23^ At the $3.20 poverty line, the picture becomes less clear and varies across the sample, while at the top poverty line there is an increase in poverty except in Sri Lanka and Uzbekistan. This reflects the fact that those around the upper poverty line are towards the top of the consumption distribution in most of these countries and would actually lose more from the abolition of preferential VAT rates than they gain from the UT. Sri Lanka and Uzbekistan have higher mean incomes than the other four countries in the sample and thus individuals with consumption close to the highest international poverty line emerge as net beneficiaries in these countries, on average. For the poverty gap – which measures the fraction of national consumption needed to pull everybody up to that poverty line – the reform reduces poverty in all countries at each poverty line.

In comparison to existing cash transfer schemes the effect of the modelled reform is also in general large. For instance, the impact on extreme poverty is much greater in Ethiopia, Ghana and Zambia than the effect of existing cash transfers, largely due to the incomplete coverage of existing schemes. In Senegal and Sri Lanka effect sizes are more similar, whereas the existing broader VAT base (and hence reduced scope for further base broadening) and more generous cash transfer scheme in Uzbekistan mean that the additional impact of the UT reform on extreme poverty is relatively small in comparison to the impact of existing cash transfers. At higher poverty lines, comparisons of the estimates for the revenue-neutral VAT and UT reform and existing cash transfer scheme are less useful, since those higher up the consumption distribution lose out, on average, in the former case.

**Figure 6.2:**
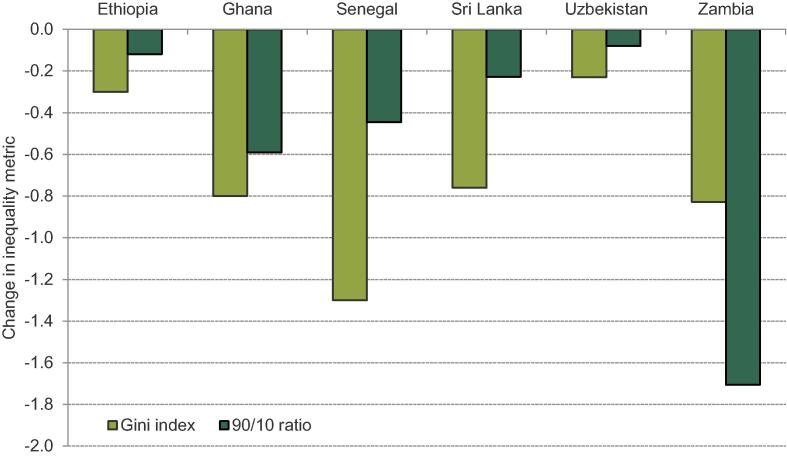
Estimates of the impact on inequality from a uniform VAT funding a UT. *Note:* Inequality measures are calculated based on consumable income per capita pre- and post-reform. *Source:* Author’s calculations using GHATAX, ETHTAX and CEQ/World Bank fiscal incidence analysis.

The pattern of results displayed for inequality metrics in Figure 6.2 are consistent. The proposal to implement a UT funded by a uniform VAT reduces both our measures of inequality in all cases. Magnitudes vary substantially though, with only a very small estimated reduction in inequality in Uzbekistan, for instance.

**Figure 6.3:**
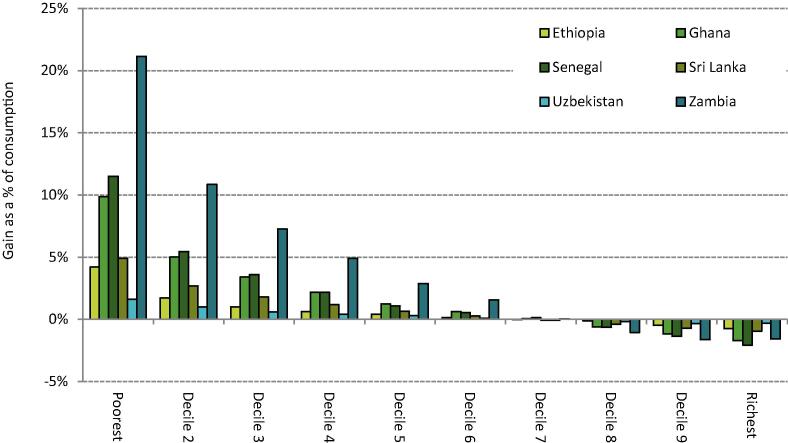
The proportion of net beneficiaries across the consumption distribution from a uniform VAT funding a UT. *Note:* Population deciles ranked by per capita consumption; an individual is counted as a net beneficiary from the reform if the increase in consumption from the UT exceeds the increase in VAT from removing exemptions and reduced rates. *Source:* Author’s calculations using GHATAX, ETHTAX and CEQ/World Bank fiscal incidence analysis.

Average distributional effects and impacts on headline poverty and inequality metrics are important but again mask heterogeneity at the household level. In this case, the heterogeneity amongst households of comparable levels of consumption is driven by differences in the extent to which those households purchase goods and services which are currently subject to preferential VAT rates. This may differ due to preferences over types of goods and services and/or the extent to which consumption comes from monetary expenditures as compared to home production and barter. Figure 6.3 provides estimates of the percentage of individuals in each consumption decile that would be net beneficiaries from the modelled VAT and UT reform. The results are as one would expect: at the bottom of the distribution almost all individuals are net beneficiaries, with the proportion of net beneficiaries largely falling monotonically as one moves up the consumption distribution. It is important to contrast these estimates with those in Figure 5.2. This comparison confirms an obvious intuition: in every country, given the structure of existing cash transfer programmes, a UT would be far better suited to ensuring widespread coverage among poor households and more effective (in terms of redistribution) at compensating for a broader VAT.

These results show a consistent picture that compared to even untargeted benefits, preferential VAT rates are an ineffective way of reaching poor households. Our analysis here has of course applied a range of simplifying assumptions, and we cannot convincingly test the robustness of our results to all of them. However, in Appendix C we offer an extension to our analysis on one important dimension for Senegal, where external data allows us to incorporate differential informality across consumption deciles and products. Doing so does not change the overall conclusions, although as expected the overall scale of redistribution from the modelled reform is reduced somewhat.

## 7 Discussion and conclusions

This paper has used microsimulation models and input–output tables to estimate the impact of preferential VAT rates on tax revenues, poverty and inequality in six LMICs (Ethiopia, Ghana, Senegal, Sri Lanka, Uzbekistan and Zambia), and compared these impacts to existing and hypothetical cash transfer programmes.

We find that preferential VAT treatments (excluding exemptions that have strong administrative justifications, such as for public services, financial services, accommodation costs and small businesses) are a poor way of channelling resources towards poor households. Although in isolation these preferential rates do reduce poverty, and potentially sometimes by a significant amount, they are very expensive and much of their benefit accrues to better off households. This is partly because monetary expenditure is relatively less important for low-consumption households (who obtain more from home production) in the LMIC context, and thus exemptions have less of an impact for such households than they would if all consumption was comprised of monetary expenditure. However, much more important is the rather banal fact that high consumption households spend more in cash terms on exempt and reduced rate goods and services. This means that the implicit cash subsidy afforded to high-consumption households is far greater, on average, and renders preferential VAT rates an expensive way of attaining a given level of poverty reduction. Although we cannot generalise this conclusion to other country contexts with certainty, the consistency of the result in the six countries studied – which have different levels of income, inequality and tax structures – leads us to believe that similar patterns should be expected in other LMICs, mirroring similar findings in the OECD and EU. Only if preferential VAT rates are targeted narrowly on goods and services consumed in greater absolute quantities by the poor would the overall qualitative conclusion be overturned.

Existing cash transfer schemes are better targeted at poor households towards the bottom of the consumption distribution. However, they may not always provide a suitable means of compensating households for the reduction in purchasing power they would face if the VAT base was broadened. Many existing schemes remain relatively small-scale and tend to target subsets of the poorest parts of the population, often based on specific demographic or geographic eligibility criteria, even before accounting for potential exclusion (and inclusion) errors. Significant increases in the scale and coverage of such schemes would be required to offset the negative impact of VAT base broadening on households with low and middling levels of consumption. In these countries, that is sometimes those below or close to the $1.90 and $3.20 international poverty lines.

A Universal Transfer (UT) would overcome coverage issues in existing cash transfer schemes. Simulating a counterfactual policy scenario in a comparable way in each of the countries considered, our results indicate that broadening the VAT base and using the revenue to fund a UT would boost the consumption of the least well-off households, reducing inequality and measures of extreme poverty in all cases. Our focus on a UT is primarily due to its simplicity and comparability across countries, allowing us to highlight the possible gains for poorer households from one policy option in different contexts. However, while governments are able to choose from a range of redistributive instruments, a UT would appear to have a number of desirable features. No sophisticated targeting would be required, in principle no vulnerable households would be excluded, and the inclusion of the middle class might be a boon to political feasibility. There are of course questions of practicality with regards to the ability of LMIC governments to administer such nationwide programmes, although the technology required is increasingly available (Ghana, for instance, has been distributing biometric ID cards to its population (Thiel, 2020)).

Moreover, there are important efficiency implications to the reforms considered in this paper. For instance, expanding the scope of VAT might increase tax evasion and shift activity into the informal sector, which would have implications for revenue yield (and the associated redistribution made possible) and productivity. Such considerations may represent one rationale for preferential VAT rates in sectors where there is deemed to be more scope to evade taxes. In addition, there are potential labour supply effects to consider, given that the reform simulated in this paper involves an increase in out-of-work (and in-work) incomes and an increase in effective tax rates, and associated general equilibrium effects. However, some evidence suggests this might not be a first-order concern in LMICs (Alzúa et al., 2013, Banerjee et al., 2017, Salehi-Isfahani and Mostafavi-Dehzooei, 2018). Indeed, one would expect labour supply distortions to be less of a concern with unconditional programmes than with means-tested benefits, since the latter induces substitution effects as well as income effects. Additionally, in LMIC contexts some induced reductions in labour supply (such as child labour) may actually be desirable (de Hoop and Rosati, 2014, Hidayatina and Garces-Ozanne, 2019). A full welfare analysis taking account of these efficiency costs alongside equity considerations would require strong assumptions given the paucity of empirical evidence on relevant elasticities in similar contexts, and thus we do not attempt to do so in this paper. These and other related issues, such as tax incidence in the presence of informal markets, are promising avenues for future research.

These considerations could impact the precise distributional patterns and magnitudes of the reforms modelled in this paper. However, the strength of our main results and the robustness checks conducted strongly suggest that the core conclusions of the paper would be robust to further extensions. In summary, there is scope for cash transfers – even when completely untargeted – to redistribute resources significantly more effectively than existing preferential VAT rates in a range of LMICs. Such reforms therefore warrant consideration – as well as further research – as countries seek to rationalise their tax systems and develop stronger social safety nets.

### CRediT authorship contribution statement

**Ross Warwick:** Conceptualization, Methodology, Software, Formal analysis, Writing – original draft, Writing – review & editing, Visualization, Supervision, Project administration. **Tom Harris:** Formal analysis. **David Phillips:** Conceptualization, Methodology, Writing – original draft, Writing – review & editing, Supervision, Project administration. **Maya Goldman:** Formal analysis, Writing – original draft, Writing – review & editing. **Jon Jellema:** Methodology, Writing – original draft, Writing – review & editing. **Gabriela Inchauste:** Conceptualization, Methodology, Writing – original draft, Writing – review & editing. **Karolina Goraus-Tańska:** Methodology, Formal analysis, Writing – original draft, Writing – review & editing.

## Declaration of Competing Interest

The authors declare that they have no known competing financial interests or personal relationships that could have appeared to influence the work reported in this paper.

## Figures and Tables

**Fig. A.C.1:**
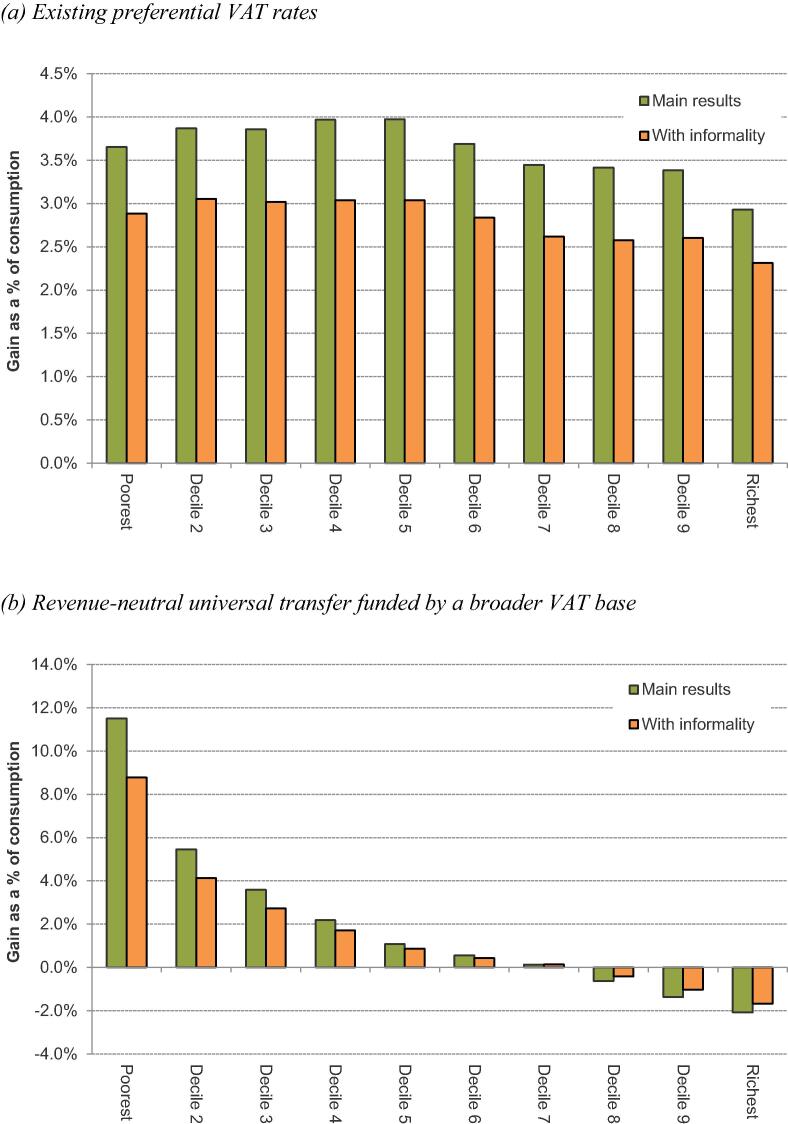
The distributional impact of existing preferential VAT rates and a UT funded by a broader VAT base in Senegal, before and after accounting for informal purchases.

**Table:** 

	**Modelled**				**Official statistics**			
	**Year**	**$1.90**	**$3.20**	**$5.50**	**Year**	**$1.90**	**$3.20**	**$5.50**
Ethiopia	2015-16	33.1	69.5	90.8	2015	30.8	68.9	90.2
Ghana	2016	8.5	26.0	53.9	2016	12.7	29.3	55.1
Senegal	2011*	41.6	71.5	90.0	2011	38.5	68.4	88.4
Sri Lanka	2016*	1.5	12.1	44.0	2016	0.9	11.0	42.0
Uzbekistan	2018	2.0	14.7	45.8	-	-	-	-
Zambia	2015	57.8	75.7	88.4	2015	58.7	75.4	88.1

## Appendix A Descriptions of VAT and cash transfer systems

### Ethiopia – Policy Year 2015–16

#### VAT system

Legislated VAT policy has a relatively wide base subject to the standard rate of 15%. Exemptions are granted on the following goods: raw cereals, soya beans, oil seeds, coffee, raw cotton, wheat flour, bread, leather products, kerosene, water purifying chemicals, mosquito nets, electricity, water, financial services, public administration services and donations, charity fees and gifts. Most exports are subject to zero-rates, except for a few for which exemptions have replaced the zero-rating classification (e.g. sesame, haricot and mung beans). There are no luxury rates, and the VAT registration threshold is 500,000 Birr in annual revenues.

63% of household consumption takes the form of monetary expenditure, while 13% of household monetary expenditure is exempt from VAT. VAT revenues contribute approximately 31% of total tax revenues collections for general government.

#### Cash transfer scheme(s)

The Productive Safety Net Programme (PSNP) is Ethiopia’s rural safety net for food insecure households, and targets households that are chronically and transitorily food insecure and provides them with cash and/or food transfers. As of June 2016, the PSNP had almost 8 million direct project beneficiaries (individuals). There are two types of transfers: conditional transfers (households that have able-bodied adult labour engage in public works and receive transfers for 6 months of the year) and unconditional transfers (households without labour capacity, permanent direct support clients, and receive 12 months of unconditional transfers while also being linked with social protection services).

The basic PSNP eligibility criteria are the following:

1) Households should be members of an eligible community (which in turn should belong to the Woreda-selected Kebeles).
2) Food insecure households or households without adequate social protection/support.

Four modes of payment are possible: 1) cash payments made using the traditional approach of WOFED cashiers; 2) Cash payments made electronically through third party payment service providers; 3) Food transfers; and 4) Food vouchers. The daily wage rate of the cash transfer is calculated on the basis of the cost of buying 3 kg of cereal and 0.8 kg of pulses per day (15 kg of cereal and 4 kg of pulses per person per month) in the market. These values vary at the Woreda-level. In practice, 61% of clients claim to receive payments within the agreed time fare, while 55% claim to receive a transfer at least equal to the value of 15 kg of cereal and 4 kg of pulses per month.

### Ghana – Policy Year 2016

#### VAT system

In essence, Ghana’s rate of VAT is 17.5%. However, 2.5% of this is labelled as a National Health Insurance Levy (NHIL). In general, zero-rating is reserved for exports; exemptions, however, are widespread and apply, for example, to many basic foodstuffs (if raw or simply prepared and domestically produced), health, education, domestic passenger transport, water, electricity, kerosene, petrol, diesel, pharmaceuticals, agricultural inputs, and textbooks. There are no “luxury” rates. Businesses whose annual turnover falls below GHS 200,000 are not required to register for VAT but may do so voluntarily.

Household survey estimates suggest that 53% of household expenditure is on goods and services that are in principle subject to VAT. Together, VAT and NHIL account for 28% of tax revenue, after accounting for refunds.

#### Cash transfer scheme(s)

The Livelihood Empowerment Against Poverty (LEAP) programme is the main cash transfer programme in Ghana and is what is modelled in this paper. LEAP began in 2008 and is a cash transfer programme providing both monetary disbursements and free national health insurance enrolment to recipient households. In 2016, 212,848 households were enrolled in the scheme.

Eligibility for the programme depends on a household being both extremely poor and having one or more member in one of the following social categories: aged 65 or above, severely disabled, orphaned or vulnerable child, or pregnant women and mothers with infants. In practice, selection for the programme depends on a community in a region being selected based on poverty data from a household survey. Individual eligible households are then identified via the use of a Proxy Means Test (PMT) to determine whether they qualify as extremely poor, as well as verification of household members in the aforementioned social categories.

The cash payments depend on the number of eligible beneficiaries in the households and are paid every second month. A household with one eligible member receives GHS 64 on each occasion; for two beneficiaries the payment is GHS 76; for three it is GHS 88 and a household with four or more beneficiaries receives GHS 106.

### Senegal – Policy Year 2015

#### VAT system

The standard rate of VAT in Senegal is 18%. Although there are no “luxury rates” above this, a reduced rate of 10% exists for accommodation and catering services owned by a licensed tourist accommodation. In addition, a wide range of goods and services are exempt from VAT including healthcare and pharmaceuticals, education, water, electricity, financial services, real estate and a list of unprocessed foods which includes cereals, vegetables, plants, roots and tubers, peanut, meat, fish and milk. There is no minimum turnover threshold for firm registration. In addition, a large list of ad hoc exemptions exists, particularly at customs, with specific firms exempt on the basis of locating in special economic zones, for instance.

In 2015, VAT accounted for 36% of total government tax revenue.

#### Cash transfer scheme(s)

The Programme National de Bourses de Sécurité Familiale (PNBSF) is the main cash transfer programme in Senegal, constituting 93% of direct transfers provided in the country. The programme was launched in 2014 and provides vulnerable families with 100,000 FCFA per year (close to 170 USD) to strengthen their livelihoods. It is a conditional cash transfer programme, with the conditionality intended to provide an incentive to keep children in school, hence strengthening households’ human capital, while parallel specific, productivity enhancing activities aim at increasing the earning capacity of the poor.

Eligibility for the programme is determined by a combination of geographic targeting, a proxy means test, and community-based targeting. In total, 19% of individuals are covered by the scheme; a recent baseline survey sampled among the Unique Registry (the universe of the CCT program) shows that more than 90% of people are poor, although only 55% are extreme poor.

### Sri Lanka – Policy Year 2017

#### VAT system

The standard rate of VAT in Sri Lanka was increased to 15% in November 2016 (as well as being temporarily at that level in May-June of that year), up from a previous rate of 11%. The only reduced rate is the standard zero-rating of exports but a wide range of goods and services are exempt from VAT – 451 product items are mentioned in the VAT act as exempt. These products include, for example, many agriculture, forestry and fishing products, textiles and leather, food products, beverages, transport services, utilities, financial services and real estate services. Estimates from household survey data suggest that about 45% of total household expenditure is on goods and services that are exempt from VAT. VAT registration is compulsory for persons whose supply of taxable goods and services exceeds LKR 12,000,000 annually (or LKR 3,000,000) per quarter are required to register for VAT.

VAT is an important source of tax revenue in Sri Lanka, contributing Rs 283 billion in 2016, equivalent to 19% of total revenue collected. The increase in the standard rate in 2016, along with a reduction in the number of exemptions, caused VAT revenue to jump substantially in 2017, up to 27% of total revenue.

#### Cash transfer scheme(s)

The main cash transfer programme in Sri Lanka is called Samurdhi and began in 1994. In 2016, there were 1.4 million beneficiary families in the programme, a sizeable decrease from a peak of 2 million in 2006. The Samurdhi programme has a number of components: an income support scheme which grants a monthly income depending on the number of children in the household, an insurance scheme providing cash during births, deaths and hospitalisations, and cash transfers for other social development programmes. Payments from the income support scheme are varied according to household size. Those with fewer than 3 members receive LKR 2,250 per month, those with 3 members receive LKR 3,600, and those with 4 or more members receive LKR 4,500.

Eligibility is determined by income: those with household income below LKR 3,000 per month are able to apply. However, income is self-reported and as such the selection of beneficiaries is ultimately at the discretion of the Divisional Secretariat and Grama Niladari-level program officers.

### Uzbekistan – Policy Year 2018

#### VAT system

The statutory VAT rate is 20%, with no higher rates. As measured by household purchases in the household survey, more than three-quarters of household consumption is subject to the standard rate. However, exemptions apply for a limited set of goods and services such as some education services, devices and facilities for disabled people, postmarks and envelopes, municipal passenger transport, precious metals and agricultural products produced domestically. The retail and wholesale sector are also exempt, while gas and water for household use are zero-rated. Compulsory VAT registration is unusually determined by number of employees rather than sales, with registration compulsory for firms with more than 200 employees.

### VAT accounted for 39% of total tax revenue in 2018

#### Cash transfer scheme(s)

We include the low-income family allowance, which comprises three arms: the childcare allowance for ages 0–2; the children allowance for ages 3–13; and the financial assistance programme. The number of beneficiary households for each programme in 2018 was 284,000, 223,000 and 61,000, respectively. For each of these, those with income below 1.5 times the minimum wage are eligible – this equated to 0.26 million so’ms per month until October 2018.

Monthly payments total 0.34 million so’ms for the childcare allowance. For the children allowance, payments equated to 0.09, 0.14 and 0.2 million so’ms for 1, 2 and 3 or more children, respectively. The financial assistance programme is available for low-income families with vulnerable children, such as those with disabilities, and provides 0.26–0.52 million so’ms per month depending on complexity.

### Zambia – Policy Year 2015

#### VAT system

The statutory VAT rate for standard-rated goods and services is 16%. There are no rates above the statutory rate but a zero rate does apply to a range of goods, including building supplies, mosquito nets, medical supplies, educational materials, energy saving equipment, and wheat flour and bread. Most other food products, health, education and housing are exempt. Firms whose turnover exceeds K800,000 in any twelve consecutive months or K200,000 in any consecutive three months are required to register for VAT; below this threshold registration is voluntary.

### In 2015, VAT accounted for 31% of total tax revenues collected by the government

#### Cash transfer scheme(s)

The main cash transfer programme is the Social Cash Transfer Scheme (SCTS), which started in 2003. By 2015 over 150,000 SCTS beneficiaries could be found in approximately 50% of the country’s 103 districts. It is an unconditional cash transfer programme involving monthly transfers of K70 for households without disabled members, and K140 for households with disabled members (approximately $US7–14, respectively).

The eligibility criteria targets poor households that include one or more disabled members and poor and vulnerable individuals. To identify eligible households, SCTS employs both a proxy means test and categorical targeting as well as community-level advice concerning potential beneficiaries. In 2017 the government was hoping to expand to cover 500,000 beneficiaries in all 103 districts as well as increasing the monthly transfer amounts.

## Appendix B Data sources

*Ethiopia*

Ethiopian Socioeconomic Survey 2015/16

Household Consumption Expenditure Survey 2015/16

Ethiopia Social Accounting Matrix 2005/06

Ethiopian Ministry of Finance Accounts

*Ghana*

Ghana Living Standards Survey Round 6 2012/13

Ghana Social Accounting Matrix 2005

Ghanaian Ministry of Finance Accounts

LEAP PMT score data for GLSS6

*Senegal*

Enquête de Suivi de la Pauvreté au Sénégal 2011

Senegal Social Accounting Matrix 2011

Senegal Ministry of Economy, Finance and Planning Accounts

*Sri Lanka*

Sri Lanka Household Consumption and Expenditure Survey 2016

Sri Lanka Input-Output Table 2011

Sri Lanka Inland Revenue Performance Reports

*Uzbekistan*

Listening to the Citizens of Uzbekistan Household’s Survey 2018

Uzbekistan Input-Output Table 2015

Consolidated State Budget of the Republic of Uzbekistan, 2013–2019

*Zambia*

Zambian Living Conditions Monitoring Survey 2015

Zambia Social Accounting Matrix 2007

Zambian Ministry of Finance Accounts

## Appendix C Accounting for informal purchases

In this Appendix, we offer additional analysis that incorporates informal purchases into our framework for Senegal. As a robustness check on our main results, we provide adjusted estimates of the distributional effect of existing preferential VAT rates, and the scale of redistribution implied by the reform scenario modelled in Section 6.

We incorporate informality into our analysis using place of purchase data. Although information on place of purchase is not directly available in any of the household surveys that underlie our main results, Bachas, Jensen and Gadenne (2021) use a different household survey dataset in Senegal which does provide this information – Enquête sur les Dépenses des Ménages de la Capitale (2008). Utilising their approach to defining informality, we match average informality at the consumption decile-by-product level from the 2008 survey into our 2011 dataset. Specifically, we assume that consumption is ‘informal’ – in this context, meaning purchased from a seller who is not registered for VAT – if it is non-market (e.g. barter or home production), from non brick-and-mortar stores (e.g. street stalls) or from corner and convenience stores. We match products across the surveys using 2-digit COICOP codes (Level 1: Divisions), creating estimates of formal and informal spending on each good by each household, where the informality share is allowed to vary by product and per-capita consumption decile.

Incorporating informality at the final stage requires a modification to the effective tax rate calculations set out in Section 3.A, in order to estimate separate vectors of effective VAT rates applying to the formal and informal sectors. Since we incorporate informality using data on where consumers make their purchases, rather than in the production process, our adapted set-up is designed to reflect the fact that informal producers at the final stage do not charge VAT on their purchases, but also cannot reclaim any VAT paid on their direct inputs. We assume in effect that products sold by formal and informal sellers follow the same formal production process up until the final stage. Since we now use data to estimate informality, we no longer calibrate it at an aggregate level, and thus the vector of effective VAT rates on domestic, formal production is given by:

$$T=\left(t-\left(E{}^{\circ}\left(t\Gamma^{D}\right)\right)+t\Gamma^{F}-\left(E{}^{\circ}\left(t\Gamma^{F}\right)\right)\right){\left(I_{N}-\Gamma^{D}\right)}^{-1}$$

The effective VAT rate paid by consumers on formal sector purchases is then given by the vector $\tau_{A}$, a weighted average of the domestic rate, and the statutory rate paid on imports.

$$\tau_{A}=\left(1_{N}-\beta \right){}^{\circ}T+\beta {}^{\circ}t$$

The effective VAT rate paid on informal sector purchases is a weighted average of the domestic informal rate and the statutory rate paid on imports, and is given by $\tau_{S}$:

$$\tau_{S}=\left(1_{N}-\beta \right){}^{\circ}\left(T+\left(E{}^{\circ}t\Gamma \right)-t\right)+\beta {}^{\circ}t$$

This vector accounts for the fact that no statutory VAT is paid on informal sector purchases at the final stage, but also that VAT paid on direct inputs cannot be reclaimed – even if the product itself is subject to VAT in principle (the vector E here is needed to ensure that embedded VAT is not added on twice for exempt products). One implication of this set-up is that we assume that informal and formal purchases of a given product are equally likely to be imported vs domestically produced. In practice this might not be true but we do not have data to vary this assumption.

Fig. A.C.1 contrasts estimates for Senegal after accounting for differential informality across products and consumption deciles with the method used for the main results in the paper. For the status quo VAT system, we underestimate actual VAT liabilities with this approach after accounting for aggregate under-recording of consumption. Thus, all VAT liabilities are scaled by a factor of 1.49 for all households in each scenario. Considering first the distributional impact of existing preferential VAT rates (panel (a)), this adjustment to our methodology suggests that informality may well be expected to reduce the revenue yield of a broader VAT base – all deciles benefit less from preferential VAT rates when we allow informality to vary across households and products compared to in our main results, where informality is assumed constant everywhere. This is driven by higher rates of estimated informality, on aggregate, for products with preferential VAT rates compared to standard-rated ones. However, informality changes the progressivity of preferential VAT rates little. Partly, this reflects that informal purchases are high, on average, across the distribution – they account for 88% of purchases in the bottom decile, and 83% in the top decile. The mild distributional pattern that does exist in terms of informality is attenuated in this case by the fact that the exempt products consumed relatively more by poorer households are more likely, on average, to be imported products – this implies a larger benefit from exemptions in our framework, which assumes full VAT compliance on imported goods.

In panel (b), the same reform as in Section 6 is modelled after accounting for informality. Households across the distribution now lose out less from a broader VAT base, and the fact that the revenue yield is lower means that the size of the UT modelled is smaller than in the main results of the paper. As a result, the overall scale of redistribution implied by the reform scenario is reduced: the poorest households gain less, and the richest households lose less, on average. However, the overall conclusion remains clear: given the only weak progressivity of existing preferential VAT rates – even after accounting for differential formality across products and households – the scope for more effective redistribution through even untargeted cash transfers remains large.

## Appendix D Poverty measures

Table A.D.1. Comparison of poverty headcount estimates in models and in official statistics.*Note:* Recent data unavailable for Uzbekistan. Modelled years shown here for Senegal and Sri Lanka differ to policy years for analysis as more recent official poverty statistics are not available; thus, we benchmark poverty rates against the latest year of official statistics in these cases. Differences can arise between modelled estimates and official statistics for many reasons, including: data cleaning choices, treatment of consumption growth between data collection and modelled year, data used for exchange rate conversion, and treatment of durable expenditure, for instance. Modelled poverty rates are based on a consistent definition of “consumable income”, described in Section 3.A.

*Source:* Modelled estimates are authors’ calculations based on data in Appendix B and World Bank data on inflation, GDP growth and PPP conversion. Official statistics are from World Development Indicators.

## Footnotes

1 Other studies in India (Roy, Raychaudhur and Sinha, 2010) and Lebanon (Salti and Chaaban, 2010), for instance, have considered the poverty and inequality implications of VAT and VAT rate increases more generally in specific LMICs, while a different perspective on VAT and inequality can be found in Alavuotunki, Haapenen and Pirttilä (2019), who study the relationship between the adoption of VAT and measures of inequality using cross-country data.

2 For example, if certain goods and services are subject to greater tax evasion, are difficult to value, or are linked to labour supply decisions.

3 See, for example, Gentilini and Omamo (2011) for a discussion of key considerations.

4 In 2016 – the policy year that we model in this paper – Ghana’s overall 17.5% rate consisted of a 15% VAT and a 2.5% tax known as the National Health Insurance Levy (NHIL), which was effectively a second VAT applying to the same tax base as the general 15% rate. Since 2017, Ghana’s VAT system has undergone a number of changes, including NHIL becoming irreclaimable, as well as a new irreclaimable levy (the GETFund Levy) in place of a further 2.5% of the original VAT.

5 Although zero rates are sometimes applied to firms that operate in special economic zones or have certificates entitling them to apply a zero rate.

6 In the case of Uzbekistan we combine three low-income family allowances: a children allowance for households with children below 14 years old, a child-care allowance for households with children below two years old, and financial assistance for households with specific needs (e.g. disability).

7 Specifically, our measure of welfare is consumption per capita minus indirect taxes plus indirect subsidies. This is equivalent to consumable income following the Commitment to Equity approach (Lustig, 2018) if consumption is assumed equal to disposable income. Consumption comprises purchased consumption goods, the value of home production, imputed rents and user values from durable goods. The exception to this is Ethiopia, where durable expenditure is included and imputed rents are not available.

8 We briefly discuss how different VAT incidence assumptions – on labour and/or capital – could affect our results in Section 4. It is worth noting that the studies cited here are based on high income countries, and findings from these will not necessarily carry over to economies with large informal sectors. In particular, one possibility is that formal sector firms would be relatively less able to pass the VAT on to consumers in the presence of informal firms not charging VAT. The incidence of sales taxes in LMICs is as yet unaddressed in the literature but is the subject of a number of ongoing studies.

9 Our approach differs to that in Coady (2008) (see also Inchauste and Jellema (2018)) in relation to our treatment of VAT incidence on domestically produced products subject to competition from imports. They assume that the producer (pre-VAT) prices of such products are determined by world prices and any unrecoverable input VAT is borne by labour, capital or land. The drawback of that approach is that unless one has information on who provides the labour, land, and capital to producers of such products, and are willing to assume (or can estimate) how the incidence of unrecoverable input VAT is shared between these factors, one cannot account for all VAT in distributional analyses. Our assumption of full incidence on consumers allows us to estimate the full (static) distributional impact using household expenditure data and input–output tables only.

10 An alternative and even more flexible approach would be to replace T, t and E with N x N matrices. This would allow for the effective and statutory VAT rates and the reclaimability of input VAT to vary not just by product i, but by each input–output linkage between products: i.e. the effective and statutory VAT rate on product *i* could vary by the product *j* for which it is being used as an input; and the reclaimability of VAT paid on inputs into the production of product *k* could vary by input product *l.*

11 Unless the production of a product involves negative value-added and a large fraction of inputs are subject to VAT.

15 As previously noted, some preferential VAT rates may be in place for reasons other than redistribution or administrative efficiency. However, typically an official policy rationale is not available in our contexts. Thus, we simulate the abolishment of the majority of preferential rates in each country.

16 This is because to the extent that abolition of preferential rates of VAT would lead to consumers engaging in substitution between different products, it would either be towards goods facing the same VAT rate (those on which the standard rate of VAT already applies) or a lower VAT rate (those products on which exemptions would still apply for administrative reasons, or on which VAT is evaded), weakly reducing the revenue yield from abolition.

17 A pro-poor transfer or tax expenditure declines in per capita terms with income or consumption.

18 The marginal effect is simply the difference between a given measure of poverty measured under two different fiscal systems.

19 These measures are also known as FGT0 and FGT1 in the class of Foster-Greer-Thorbecke indices.

20 Note that the estimated marginal effects on poverty depend a great deal on baseline poverty rates, which differ substantially across countries (see Appendix D). Thus, the marginal effects on poverty of exemptions and reduced rates are larger at lower poverty lines in poorer countries and higher poverty lines in relatively better-off countries.

21 We use the former term as the transfer that we model is not a ‘basic income’ in any meaningful sense, as indicated by the figures in the third column of Table 4.2.

22 See Banerjee, Niehaus and Suri (2019) for a review of schemes and issues to consider in the developing world.

23 The poverty rates underlying these calculations are based on measures produced from the datasets underlying our models, using a harmonised method across countries which means poverty levels do not always accord with official statistics. See Appendix D for a comparison of official statistics against the estimates generated by our models.

## Footnotes

12 Note that we could have alternatively applied a weighted average of VAT rates and ability to reclaim input VAT within a category of the input–output table to arrive at the same result.

13 Full details available from the authors on request.

14 More detailed input–output tables and SAMs that distinguish between domestically-produced and imported inputs by product category are available for a number of developed and larger developing countries (see for instance, Timmer et al (2015)). If this data were available, it would clearly be preferably to assuming that all sectors use the domestic and imported varieties of each commodity in the same proportions.
